# Dopaminergic Modulation of Signal Processing in a Subset of Retinal Bipolar Cells

**DOI:** 10.3389/fncel.2020.00253

**Published:** 2020-08-14

**Authors:** Chase B. Hellmer, Jeremy M. Bohl, Leo M. Hall, Christina C. Koehler, Tomomi Ichinose

**Affiliations:** Department of Ophthalmology, Visual and Anatomical Sciences, Wayne State University School of Medicine, Detroit, MI, United States

**Keywords:** retina, dopamine, patch clamp, visual signal processing, temporal processing

## Abstract

The retina and the olfactory bulb are the gateways to the visual and olfactory systems, respectively, similarly using neural networks to initiate sensory signal processing. Sensory receptors receive signals that are transmitted to neural networks before projecting to primary cortices. These networks filter sensory signals based on their unique features and adjust their sensitivities by gain control systems. Interestingly, dopamine modulates sensory signal transduction in both systems. In the retina, dopamine adjusts the retinal network for daylight conditions (“light adaptation”). In the olfactory system, dopamine mediates lateral inhibition between the glomeruli, resulting in odorant signal decorrelation and discrimination. While dopamine is essential for signal discrimination in the olfactory system, it is not understood whether dopamine has similar roles in visual signal processing in the retina. To elucidate dopaminergic effects on visual processing, we conducted patch-clamp recording from second-order retinal bipolar cells, which exhibit multiple types that can convey different temporal features of light. We recorded excitatory postsynaptic potentials (EPSPs) evoked by various frequencies of sinusoidal light in the absence and presence of a dopamine receptor 1 (D_1_R) agonist or antagonist. Application of a D_1_R agonist, SKF-38393, shifted the peak temporal responses toward higher frequencies in a subset of bipolar cells. In contrast, a D_1_R antagonist, SCH-23390, reversed the effects of SKF on these types of bipolar cells. To examine the mechanism of dopaminergic modulation, we recorded voltage-gated currents, hyperpolarization-activated cyclic nucleotide-gated (HCN) channels, and low-voltage activated (LVA) Ca^2+^ channels. SKF modulated HCN and LVA currents, suggesting that these channels are the target of D_1_R signaling to modulate visual signaling in these bipolar cells. Taken together, we found that dopamine modulates the temporal tuning of a subset of retinal bipolar cells. Consequently, we determined that dopamine plays a role in visual signal processing, which is similar to its role in signal decorrelation in the olfactory bulb.

## Introduction

Continuous integration of our sensory perceptions gives rise to our daily experience of the world, and this experience is made possible by specialized neuronal “antennae,” such as the retina and the olfactory bulb. Interestingly, the retina and the olfactory bulb utilize similar neural network architecture despite processing different signals. In both systems, sensory signals stimulate sensory receptor neurons, which facilitate information transfer through specific networks formed by interneurons and then project to the cerebral cortex by output neurons.

In the retina, rod and cone photoreceptors transduce light inputs into electrochemical signals. Second-order neurons, bipolar cells, converge photoreceptor input, and begin the process of extracting abstract visual features such as luminance, contrast, chromaticity, and spatiotemporal properties of light signals. This information is modulated by horizontal and amacrine cells and relayed to third-order retinal ganglion cells (RGCs), the output neurons of the retina (Wässle, 2004; Dowling, 2012). Similarly, in the olfactory system, olfactory receptor neurons (ORNs) in the nasal cavity convey odorant signals to the glomeruli structures in the olfactory bulb, where signals are modulated by juxtaglomerular cells before being sent out to the olfactory cortex by the olfactory output neurons, mitral and tufted cells (Astic et al., 1987; Stewart and Pedersen, 1987; Ressler et al., 1994; Gire et al., 2012).

Besides similarities in circuitry, the visual and olfactory processing systems also comparably use dopamine as a principal neuromodulator. Five types of dopamine receptors have been identified and are classified as D_1_-like (D_1_ and D_5_ receptors) and D_2_-like (D_2_, D_3_, and D_4_ receptors) receptors. D_1_-like receptor signaling stimulates a protein kinase A (PKA) pathway in which cAMP is increased, while D_2_-like receptor signaling decreases cAMP levels (Witkovsky, 2004; Iuphar, 2020). In the olfactory bulb, dopamine facilitates a gain control system, which presynaptically suppresses ORN transmission to the glomeruli, and also decorrelates the signals between glomeruli (Wachowiak and Cohen, 1999; Banerjee et al., 2015; Vaaga et al., 2017). Consequently, dopamine neuromodulation mediates odor signal discrimination, as shown by behavioral studies (Kruzich and Grandy, 2004; Tillerson et al., 2006; Wei et al., 2006).

In the retina, dopamine is released by dopaminergic amacrine cells (DACs) in response to light or circadian time (Kramer, 1971; Iuvone et al., 1978; Pourcho, 1982; Mariani and Hokoc, 1988; Kirsch and Wagner, 1989; Witkovsky et al., 1993; Weiler et al., 1997; Megaw et al., 2006). Retinal dopamine enables the retina to adapt from dark to daytime light conditions and has accordingly been shown to lower the light sensitivity across photoreceptor, horizontal cell, and RGC populations as light levels increase (Jensen and Daw, 1986; Vaquero et al., 2001; Hayashida and Ishida, 2004; Hayashida et al., 2009; Blasic et al., 2012; Ogata et al., 2012; Liu et al., 2016b; Nikolaeva et al., 2019). Furthermore, retinal dopamine uncouples horizontal cell and amacrine cell gap junctions with light adaptation, narrowing the receptive field size of downstream neurons (McMahon and Mattson, 1996; He et al., 2000). However, whether dopamine contributes to visual signal decorrelation—much like in the olfactory system—remains unclear.

Previously, our lab has shown that subsets of retinal bipolar cells express the dopamine receptor D_1_ (D_1_Rs) in a type-dependent manner (Farshi et al., 2016). Bipolar cells are known to perform parallel processing in the visual system (Wässle, 2004), where each type conveys distinct aspects of image features (Borghuis et al., 2013; Euler et al., 2014; Ichinose et al., 2014; Ichinose and Hellmer, 2016). Therefore, we examined whether dopamine signaling differentially modulated visual signaling in each type of bipolar cell. As the retina and olfactory bulb have extensive similarities in their signal processing, including the general role of dopamine in neuromodulation and sensory discrimination, a deeper understanding of dopamine’s function in the retina may translate to more significant insights into olfactory processing and vice versa.

## Materials and Methods

### Ethical Approval

All animal procedures were approved by the Institutional Animal Care and Use Committee at Wayne State University (protocol no. A05-03-15). All the necessary steps were taken to minimize animal suffering. The tissues were harvested immediately after the animal was euthanized by CO_2_ inhalation and cervical dislocation.

### Retinal Preparation

The experimental techniques were similar to previously described (Ichinose and Lukasiewicz, 2012; Ichinose et al., 2014). Briefly, the mice (4–12 weeks old; male or female, C57BL/6J strain; Jackson Laboratory, Bar Harbor, ME, USA or Kcng4-cre strain; a gift from Dr. Sanes, Harvard University, Cambridge, MA, USA; Duan et al., 2014) were dark-adapted overnight, euthanized, and eyes were enucleated. Using a stereo microscope, the retina was isolated and cut into slice preparations (250 μm thick). Some ganglion cell recordings were conducted using the wholemount retinal preparations. Only the dorsal retina was used for recordings. All procedures were performed in dark-adapted conditions under infrared illumination using infrared viewers. The dissecting medium was cooled and continuously oxygenated. Retinal preparations were stored in an oxygenated dark box at room temperature.

### Whole-Cell Recordings

Whole-cell patch-clamp recordings were made from the bipolar cell or ganglion cell somas in the retinal preparations by viewing them with an upright microscope (Slicescope Pro 2000, Scientifica, UK) equipped with a CCD camera (Retiga-2000R, Q-Imaging, Canada). The light-evoked postsynaptic potentials and currents (L-EPSPs and L-EPSCs) were recorded at the resting membrane potential and the equilibrium potential for chloride ions (E_Cl_; −60 mV), respectively. All recordings were performed at 30–34°C. The liquid junction potentials were corrected after each recording. Whole-cell recordings from bipolar cells usually lasted 20–30 min without significant rundown (Ichinose et al., 2014). The electrodes were pulled from borosilicate glass (1B150F-4; WPI, FL, USA) with a P1000 Puller (Sutter Instruments, Novato, CA, USA) and had resistances of 8–12 MΩ. Clampex and MultiClamp 700B (Molecular Devices, San Jose, CA, USA) were used to generate the waveforms, acquire the data, and control light stimuli by a light-emitting diode (LED; Cool LED, UK). The data were digitized and stored on a personal computer using Axon Digidata 1440A (Molecular Devices). The responses were filtered at 1 kHz with the four-pole Bessel filter on the MultiClamp 700B and sampled at 2–5 kHz.

### Solutions and Drugs

The retinal dissections were performed in HEPES-buffered extracellular solution containing the following (in mM):115 NaCl, 2.5 KCl, 2.5 CaCl_2_, 1.0 MgCl_2_, 10 HEPES, and 28 glucose, adjusted to pH 7.4 with NaOH. Physiological recordings were performed in Ames’ medium buffered with NaHCO_3_ (Millipore–Sigma, St. Louis, MO, USA) and bubbled with 95% O_2_ and 5% CO_2_; the pH was 7.4 at 30–33°C. The intracellular solution contained the following (in mM):111 potassium gluconate, 1.0 CaCl_2_, 10 HEPES, 1.1 EGTA, 10 NaCl, 1.0 MgCl_2_, 5 ATP-Mg, and 1.0 GTP-Na, adjusted to pH 7.2 with KOH. The potassium gluconate was replaced with cesium gluconate for the recording in voltage-clamp mode. A cocktail of inhibitory receptor antagonists, including a glycine receptor antagonist, strychnine (1 μM, Sigma), a GABA_A_ receptor antagonist, (−)-bicuculline methobromide (50 μM; Axxora, NY, USA), and a GABA_C_ receptor antagonist, (1,2,5,6-tetrahydropyridin-4-yl) methylphosphinic acid hydrate (TPMPA; 50 μm; Bio-Techne Company, Devens, MN, USA), were bath applied throughout all recordings to suppress the network effect. For pharmacological experiments either SKF-38393, a D_1_R agonist (10 μM; Bio-Techne Company, Devens, MA, USA), or SCH-23390, a D_1_R antagonist (10 μM; Bio-Techne Company, Devens, MA, USA), were bath applied. SKF-38393 was applied alone after control recordings, and then SCH-23390 was subsequently applied alone during the washout of SKF-38393.

### Light Stimulation

**Figure 1:**
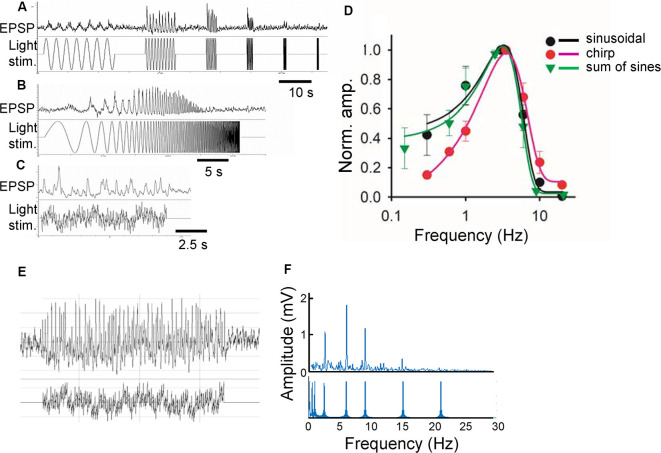
Comparison of three stimulus functions. **(A)** Sinusoidal light stimuli from 0.3 to 20 Hz (lower) and a representative response (light-evoked postsynaptic potentials; L-EPSP; upper). **(B)** Chirp light stimuli and L-EPSP with the same frequencies from the same cell. **(C)** Sum of sinusoidal light stimuli of similar frequencies and L-EPSP. **(D)** Comparison of L-EPSPs evoked by three functions (*n* = 3 ganglion layer cells). **(E)** A representative L-EPSP from bipolar cells (upper) evoked by a sum of sinusoidal light stimulation (lower). **(F)** An fast fourier transformation (FFT) analysis of the traces **(E)** revealed the different frequencies of light stimuli (lower) and the amplitude of L-EPSP for each frequency.

Green light (500 nm) was projected through a 60× objective lens onto the photoreceptors in the vicinity of the recorded bipolar cells with a spot diameter of 100 μm, which is slightly larger than the size of the receptive field center for a bipolar cell (Berntson and Taylor, 2000). The preparations were adapted to a background light at the rod-saturated level, 4.35 × 10^4^ photons/μm^2^/s for a minimum of 10 min before recording. The same average luminance was used for subsequent sinewave stimuli. A series of sinewave stimuli discloses the temporal features of a neuron (Figures 1A,D). However, this stimulus requires a long recording time, which hampers stable pharmacological experiments. Therefore, two other equivalent light stimuli were compared: chirp and sum-of-sines (a series of stimulus takes 90 s for individual sines, 30 s for chirp, and 20 s for sum-of-sines). The chirp and sum-of-sines stimuli evoked generally similar light responses compared to the original stimulus, but the sum-of-sines better replicated the results, especially at low frequencies (Figures 1B–D). A representative bipolar cell sum-of-sines recording and resulting power spectrum are shown (Figures 1E,F).

### Voltage-Gated Channel Recording

Hyperpolarization-activated cyclic nucleotide-gated (HCN) channels and low-voltage activated (LVA) Ca^2+^ channels were recorded by voltage-clamp mode. HCN currents were activated by hyperpolarization (–60 to –130 mV) for 1 s followed by holding the potential at –70 mV (Figure 5A; Cangiano et al., 2007; Hellmer et al., 2016). Voltage-gated Ca^2+^ channels were evoked by a ramp voltage change from –90 to +44 mV at a speed of 134 mV/s (Hu et al., 2009). HCN currents were isolated based on our previous pharmacological experiments (Hellmer et al., 2016). LVA currents were also isolated by including potassium channel blockers (Cs and TEA) in the pipette solution (Hu et al., 2009).

**Figure 2:**
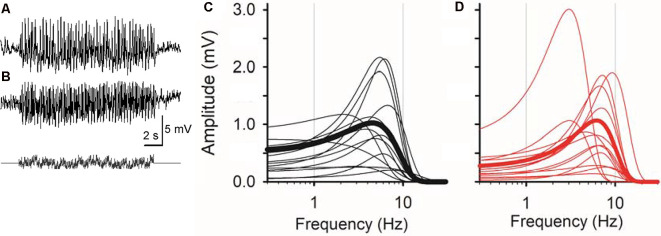
Sinusoidal L-EPSPs were changed by dopamine receptor 1 (D_1_R) signaling in bipolar cells. **(A)** A representative L-EPSP recorded from an XBC cell in response to a sum-of-sines light stimulus (bottom). **(B)** L-EPSPs in the presence of 10 μM SKF38393. **(C)** Bipolar cell temporal responses were fit to the exponential curves. Each curve exhibits a frequency-response relation for each bipolar cell (black) and the average population response (thick black) in the control solution. **(D)** The temporal responses for each bipolar cell (red) and the average response (thick red) in the presence of 10 μM SKF38393. The peak frequency response shifted to the right in some bipolar cells, indicating that D_1_R signaling increased the sensitivity to higher frequencies.

**Figure 3:**
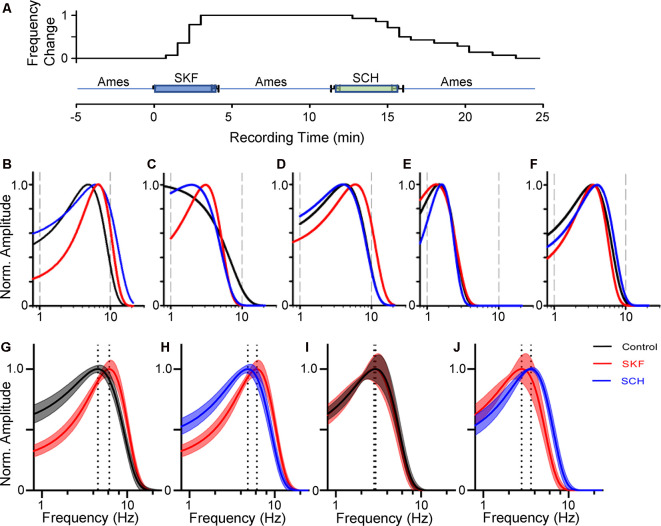
Dopamine receptor 1 (D_1_R) signaling increased the peak temporal tuning in some bipolar cells. **(A)** The time course of SKF-SCH applications and the normalized peak frequency change. The peak frequency is indicated 0 at the control level, and 1 when shifted to a higher frequency. The plot shows the individual response from the 14 bipolar cells. **(B–D)** The frequency-response curves for a transient OFF **(B)**, sustained OFF **(C)**, and transient ON **(D)** SKF sensitive bipolar cell in control (black), SKF (red), and SCH (blue). SKF showed an increase in the peak temporal frequency for all three of the cells. Subsequent SCH application reduced the peak temporal frequency. **(E,F)** The frequency response curves for an OFF **(E)** and ON SKF insensitive bipolar cell in control (black), SKF (red), and SCH (blue) conditions respectively. SKF or SCH application did not change the temporal tuning for these two cells. **(G)** The average frequency-response curves from 14 bipolar cells (including ON and OFF cells) in control (black, average ± SEM), and in the presence of SKF (red). The peak frequencies are indicated by dotted lines. SKF shifted the peak to the right, indicating that D_1_R signaling increased the temporal tuning for these cells towards higher frequencies. **(H)** The frequency-response curves from the same set of cells in the presence of SKF (red) and subsequent SCH (blue) application. SCH shifted the curve back to the control level. **(I)** The average frequency-response curves from six bipolar cells in control (black) and SKF (red) solutions. SKF did not change the curve for these bipolar cells. **(J)** The curves from the same set of bipolar cells in SKF (red) and SCH (blue) solutions.

**Figure 4:**
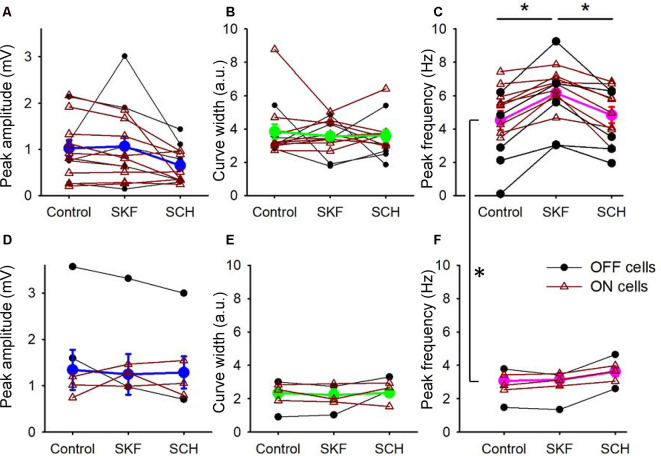
D_1_R signaling modulated the peak frequency, but not for the peak amplitude and the tuning curve width. **(A)** The peak amplitude of the frequency-response curves for the SKF-sensitive bipolar cells. Each dot-line represents one cell, and the average is shown in thick blue. ON and OFF cells are color-coded in brown and black, respectively. Both SKF and SCH did not change the peak amplitude. **(B)** The tuning curve width of the frequency response curves for the SKF-sensitive bipolar cells. Individual cells (brown and black) and the average (green). Neither SKF nor SCH changed the average tuning curve widths. **(C)** The peak frequency of the frequency-response curves for the SKF-sensitive bipolar cells. Individual cells are shown in brown and black, and the average is shown in magenta. SKF significantly increased the peak frequency (*p* < 0.01), which was reversed by SCH application (*p* < 0.01). **(D–F)** The same set of parameters for SKF-insensitive bipolar cells. The D_1_R agonist and antagonist changed none of the three parameters. **(C,F)** The peak frequencies in control solutions for the SKF-sensitive and -non-responsive bipolar cells are significantly different (*p* < 0.01, *p < 0.05).

**Figure 5:**
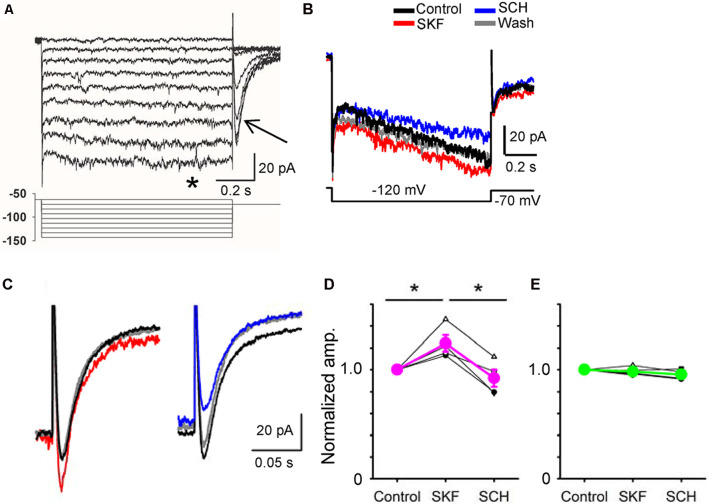
A D_1_R agonist and an antagonist modulated hyperpolarization-activated cyclic nucleotide-gated (HCN) currents in a bipolar cell. **(A)** Voltage steps (lower panel) evoked inward currents in a type 5-2 bipolar cell. Steady-state currents (*) and tail currents (arrow). **(B)** HCN currents were evoked in a type 5-2 bipolar cell. SKF increased the HCN steady-state current, whereas SCH decreased the current. **(C)** The tail current was also increased by SKF38393 (red). SCH23390 decreased the tail current (blue) in the same cell. **(D)** A summary graph shows that SKF increased the HCN tail current in four bipolar cells (*p* < 0.05), and SCH decreased the current (*p* < 0.01, *p < 0.05.). **(E)** In five bipolar cells, SKF and SCH did not change the HCN tail current (*p* > 0.1).

### Morphological Identification

A fluorescent dye, sulforhodamine B (0.005%, Sigma), and Neurobiotin (0.5%, Vector Lab, Burlingame, CA, USA) were included in the patch-clamp pipette. Immediately after electrophysiological recordings, sulforhodamine B images were captured using the CCD camera. For Neurobiotin visualization, the slice preparation was fixed with 4% paraformaldehyde for 30 min, incubated with streptavidin-conjugated Alexa 488 (1:200, Thermo Fisher Scientific, Waltham, MA, USA) and an anti-choline acetyltransferase (ChAT) antibody (1:200, AB144P, Millipore, Danvers, MA, USA) overnight, and then incubated with the secondary antibody for 2 h at room temperature. The preparation was viewed with a confocal microscope (TCS SP8, Leica, Germany). We determined bipolar cell types according to previous descriptions (Ghosh et al., 2004; Ichinose et al., 2014; Ichinose and Hellmer, 2016).

### Data Analysis and Statistics

For sinusoidal responses, MatLab (MathWorks, MA, USA) and pClamp were used to measure amplitude (in mV) by Fast Fourier Transformation (FFT) analysis. Fundamental and multiple harmonics amplitudes were added to achieve accurate amplitude measurements. After FFT analysis, the frequency-responses were fitted with curves using the equation:

$$Y=a*e{\left(-0.5*(x-x_{0})/b\right)}^{2}$$

Where *a* = peak amplitude, *b* = tuning curve width, *x*_0_ = frequency at the peak. Hereafter, we refer to *x*_0_ as the peak frequency or the frequency where cells responded with the largest response amplitude. Correspondingly, the peak amplitude indicates the amplitude response at the peak frequency. Finally, tuning curve width indicates the range of frequencies over which the cell responds, and is equivalent to the full width at half-maximum (FWHM) variable reported in other literature.

For HCN recordings, the tail current amplitude was analyzed to decrease contamination from other currents (Horwitz et al., 2011; He et al., 2014). The values are presented as the mean ± SEM. Voltage-gated currents and L-EPSCs were normalized to the control level because of large variations of individual currents between cells. A repeated-measures ANOVA was conducted to compare the response in control, SKF, and SCH solutions (Prism v.8, GraphPad Software, CA, USA). The ANOVA was run with a Geisser-Greenhouse correction to account for possible violations of the assumption of circularity/sphericity, followed by a Tukey’s multiple comparisons test to obtain the adjusted *p*-values. A paired *t*-test was conducted to compare the voltage-gated currents in control and SKF solutions. The differences were considered significant if *p* < 0.05.

## Results

### D_1_R Signaling Modulates Temporal Features in a Subset of Bipolar Cells

Whole-cell recordings were conducted from bipolar cells using retinal slices prepared in the dark under infrared illumination. After adapting the preparations at a mesopic light level for more than 10 min, light-evoked excitatory postsynaptic potentials (L-EPSPs) were recorded in response to the sum-of-sines stimuli in the presence of inhibitory receptor blockers (see “Materials and Methods” section; Figure 2A). L-EPSPs for individual cells were analyzed by FFT (Figure 1), which revealed the frequencies of sinewave stimuli and L-EPSP amplitudes for those frequencies. The response amplitude was plotted as a function of frequency and fitted by an exponential equation (see “Materials and Methods” section) for each cell. The frequency-response curves from 14 bipolar cells, including representative recordings from types 3, 4, 5, XBC, and 6, are plotted in Figure 2C (black lines). The peak amplitude, tuning curve width, and peak frequency were diverse among cells because multiple types of bipolar cells exhibit distinct temporal features (Ichinose et al., 2014; Ichinose and Hellmer, 2016).

Previously, we found that the dopamine receptor 1 (D_1_R) is expressed by bipolar cells in a type-dependent manner (Farshi et al., 2016). D_1_Rs are expressed by types 1, 3b, 4, 5-2, XBC, 6, and 7 bipolar cells, whereas types 2, 3a, 5-1, and 9 did not possess the receptor. While morphological evidence has suggested that dopaminergic signaling plays a distinct role in visual processing in each type of bipolar cell, physiological investigations have not been performed. We examined the dopaminergic effect on the temporal features in bipolar cells by the application of a D_1_R agonist, SKF38393 (10 μM; Figure 2B). In some bipolar cells, SKF shifted the peak frequency to higher frequencies (Figures 2C,D, compare average traces shown by thick black and red lines, 14/20 cells), suggesting that D_1_R signaling modulated the temporal tuning.

We have examined the dopaminergic effect on temporal features in both ON and OFF bipolar cells. We recorded L-EPSPs in the control solution, applied SKF38393 (10 μM), for 4 min, washed-out approximately for 8 min, and then applied an antagonist, SCH23390 (10 μM; Figure 3A). In 14 ON and OFF bipolar cells, the peak frequencies shifted toward higher frequencies by SKF (Figures 3B–D,G), and the subsequent application of SCH moved the peak frequency back to the control level (Figures 3B–D,H). The time course of SKF-SCH applications and the responses from 14 bipolar cells (Figure 3A) shows that the peak frequencies changed during the SKF application (at 2.1 ± 0.2 min) and changed back to the control level after the SCH application (at 15.2 ± 0.8 min), indicating that D_1_R signaling modulated the temporal responses. Furthermore, we observed that SCH-only application shifted the peak frequency to lower frequencies (*n* = 2), or no change (*n* = 2), suggesting that some ambient dopamine may already be present in our light-adapted condition. In the other six bipolar cells, the peak frequency responses were not shifted by SKF (Figures 3D,E,H,I); therefore, we categorized them as SKF-insensitive cells. The former group included type 3 (*n* = 4), type 4 (*N* = 1), type 5 (*n* = 7), and type 6 (*n* = 2), whereas the latter contained type 2 (*n* = 1), type 3 (*n* = 1), type 5 (*n* = 3), and type 6 (*n* = 1). Although the number of recordings from individual types was low and no further type-specific morphological features (such as type 3a and 3b) were determined in this study, the observed cell types were consistent with our previous findings of D_1_R-expressing and non-expressing bipolar cells (Farshi et al., 2016).

We compared the parameters of frequency-response curves between SKF-sensitive and insensitive bipolar cells by repeated measures ANOVA (see “Materials and Methods” section). The peak amplitude, tuning curve width, and the peak frequency of SKF-sensitive bipolar cells were plotted (*n* = 14; Figures 4A–C). The former two factors were not affected by SKF, nor SCH. However, the peak frequency was shifted to higher frequencies by SKF (*p* < 0.01) and returned to the control level after SCH (*p* < 0.01; Figure 4C). For the SKF-insensitive bipolar cells, none of these parameters were modulated by SKF, nor by SCH (*p* > 0.1 for all combinations; Figures 4D–F). We also compared the peak frequency for SKF-sensitive ON and OFF bipolar cells separately (*n* = 9 for ON, *n* = 5 for OFF); SKF shifted the peak frequency to a higher frequency (*p* < 0.01 both for ON and OFF) and SCH decreased it (*p* = 0.05 for OFF, *p* < 0.01 for ON). Furthermore, in control conditions, the peak frequency of SKF-sensitive cells was higher than for the SKF insensitive cells (*p* < 0.01; Figures 4C,F). One possible explanation for this is that due to slicing, these cells may have been damaged in some way and therefore respond slower as a result; however, we note that the mean peak frequency of SKF-insensitive cells (2.8 ± 0.3 Hz; Figure 4F, control) are similar to those found in previous slice studies (Burkhardt et al., 2007; Ichinose and Hellmer, 2016). These results indicated that D_1_R signaling enables a subset of bipolar cells to respond to higher frequency stimuli during light-adapted conditions.

### Voltage-Gated Channels in Bipolar Cells Are Targets for Dopaminergic Modulation

The temporal properties of bipolar cells are shaped by multiple factors, including ligand or voltage-gated channel diversity, the mGluR6 complex, and amacrine cells (DeVries, 2000; Ma et al., 2003; Müller et al., 2003; Cao et al., 2012; Baden et al., 2013; Puthussery et al., 2013; Lindstrom et al., 2014; Ray et al., 2014; Franke et al., 2017). Among these factors, voltage-gated channels, such as HCN and voltage-gated Ca^2+^ channels, are also known-targets of dopamine (Pfeiffer-Linn and Lasater, 1993; Surmeier et al., 1995; Fan and Yazulla, 2001; Robinson and Siegelbaum, 2003; Hayashida and Ishida, 2004). Therefore, we investigated whether D_1_R signaling modulated hyperpolarization-activated cyclic nucleotide-gated (HCN) channels and low voltage-activated (LVA) Ca^2+^ channels in D_1_R-expressing bipolar cells.

Whole-cell recordings were conducted from bipolar cells, and HCN currents were evoked in response to a series of step pulses (Figure 5A; Cangiano et al., 2007; Hellmer et al., 2016). Steady-state and tail currents were recorded. After steady recordings were obtained, we applied SKF, followed by SCH in the bath solution. In four bipolar cells, including type 1 (*n* = 1), type 3 (*n* = 1), and type 5 (*n* = 2), SKF increased the tail current, and SCH reduced the current (Figures 5B–D, *P* < 0.05). In contrast, SKF and SCH did not affect the tail current in five other bipolar cells, including type 2 (*n* = 1), type 4 (*n* = 1), type 5 (*n* = 1), type 8 (*n* = 1), and the rod bipolar cell (*n* = 1). The SKF-sensitive bipolar cell types likely correspond with the types of bipolar cells that exhibited D_1_Rs (Farshi et al., 2016). These results suggest that D_1_R-signaling increased the peak temporal tuning in a subset of bipolar cells by increasing HCN currents.

**Figure 6:**
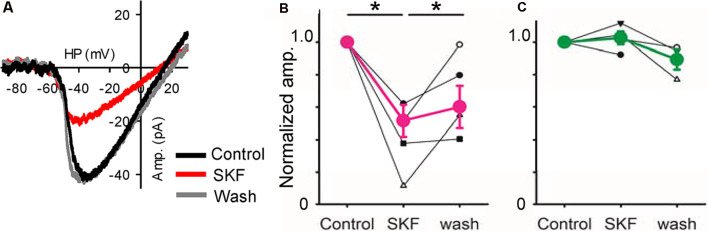
A D_1_R agonist modulated low-voltage activated (LVA) Ca^2+^ currents. **(A)** A voltage ramp evoked LVA Ca^2+^ currents in an XBC. SKF reduced it, an effect that recovered after SKF was washed from the solution clause; HP = holding potential. **(B)** Normalized amplitudes from six bipolar cells (3 type 5, 1 type 4, and 2 type 6) in response to SKF. SKF reduced the current (*p* < 0.01, *p < 0.05.). **(C)** Normalized amplitudes from four bipolar cells (1 type 5, and 3 rod-bipolar cells). SKF did not change the currents.

Furthermore, we examined the effect of D_1_R signaling on LVA Ca^2+^ channels in bipolar cells. The LVA current was evoked by a ramp voltage stimulation (Figure 6). SKF reduced the LVA currents (Figure 6A) in a subset of bipolar cells. The SKF-sensitive cells were type 4 (*n* = 1), type 5 (*n* = 3), and type 6 (*n* = 2), whereas SKF insensitive cells were type 5 (*n* = 1), and rod bipolar cells (*n* = 3; Figures 6B,C). These types were consistent with the previous morphological analysis (Farshi et al., 2016). These results suggest that D_1_R-signaling modulates LVA currents, which may modulate temporal tuning in a subset of bipolar cells.

## Discussion

Using retinal slice preparations from the mouse, we examined the effect of a D_1_R agonist and antagonist on the temporal features of bipolar cell signaling. In a subset of ON and OFF bipolar cells, the D_1_R agonist SKF38393 increased sensitivity to higher frequency responses, which was reversed by the application of a D_1_R antagonist, SCH23390. SKF38393 also increased HCN and decreased LVA Ca^2+^ currents in a subset of bipolar cells, suggesting that voltage-gated channels may be the underlying mechanism involved in D_1_R activation. The types of SKF-sensitive and SKF insensitive bipolar cells were consistent with those we previously identified as D_1_R-expressing and D_1_R-lacking bipolar cells. As the retina and olfactory bulb signal processing structures are similar, we incorporated our findings to examine similarities between retinal and olfactory dopamine neuromodulation.

### Dopamine and Signal Decorrelation in the Olfactory System

The mouse olfactory system is capable of discerning more than 10^12^ odors, resulting from over 1,000 unique odorant receptors expressed by ORNs in the olfactory epithelium (Zhang and Firestein, 2002; Bushdid et al., 2014). ORN receptors are sensitive to odorant size, shape, charge, and odorant molecule functional groups; therefore, a single odorant will activate a unique combination of odorant receptors that requires decoding by higher-order neurons (Malnic et al., 1999). Each population of unique ORNs project to distinct glomeruli in the mouse olfactory bulb, where nearly 1,800 glomeruli operate in parallel (Ressler et al., 1994; Mombaerts et al., 1996).

Within a single glomerulus, odorant information output is controlled primarily by mitral cells which balance excitation from glutamatergic ORNs and external tufted cells (Tatti et al., 2014) as well as inhibition from GABAergic periglomerular cells (PGs). To decode information from multiple odorants that may have overlapping receptor activation, glomeruli utilize lateral inhibition through another subset of GABAergic juxtaglomerular cells, the superficial short axon (sSA) cells. sSAs are excited by input within a strongly activated glomerulus and inhibit the output of other, more weakly activated glomeruli by using a combination of dopamine and GABAergic mechanisms (Aungst et al., 2003; Parrish-Aungst et al., 2007). The balance of excitation and inhibition both within and between glomeruli gates which information is sent to higher cortical areas.

Dopamine plays a critical role in odorant discrimination by contributing to the lateral inhibition between glomeruli. First, dopaminergic sSAs within a single glomerulus mediate lateral inhibition to other less-activated glomeruli in part by activating inhibitory D_2_Rs on the neighboring ORN axon terminals, reducing afferent input (Hsia et al., 1999; Ennis et al., 2001; Vaaga et al., 2017). Dopamine also indirectly inhibits mitral cell output by activating excitatory D_1_Rs in external tufted cells; external tufted cells release glutamate onto PG neurons which then locally inhibit mitral cell output (Liu et al., 2013, 2016a). Besides sSAs, dopaminergic subsets of PGs are thought to contribute to local inhibition of ORN afferent inputs, but this effect has yet to be shown directly (Maher and Westbrook, 2008). Ultimately, olfactory dopamine modulation serves to decorrelate odorant information by enhancing odorant dissimilarities and allowing the animal to discriminate multiple different odors in its environment (Wei et al., 2006; Escanilla et al., 2009; Banerjee et al., 2015).

### Dopamine and Retinal Visual Processing

In the retina, dopamine release is evoked by light stimulation from tyrosine hydroxylase (TH)-positive amacrine cells (DACs), and is thought to facilitate the transition from dim to bright ambient light conditions, such as in dawn to early morning (Krizaj, 2000; Witkovsky, 2004; Zhang et al., 2007). Therefore, dopamine is thought to contribute to light adaptation and circadian rhythm. Dopamine receptors are expressed throughout the retina; D_1_ receptors are expressed by a broad range of retinal network neurons, whereas D_2_-like receptors are present in photoreceptors and DACs (Cohen et al., 1992; Harsanyi and Mangel, 1992; Veruki and Wässle, 1996; Derouiche and Asan, 1999; Mora-Ferrer et al., 1999; Stella and Thoreson, 2000; Witkovsky, 2004). Dopamine release has a broad range of effects on retinal neurons.

Rod and cone photoreceptors have high and low light sensitivities, respectively, covering all ranges of light conditions from night to daylight. Rods and cones are furthermore coupled with homologous and heterologous gap junctions (DeVries et al., 2002; Hornstein et al., 2005). The rod-rod coupling is critical in low light conditions to integrate small inputs from multiple rods, thereby averaging signals across rods to improve the signal to noise ratio (Fain, 1975; Hornstein et al., 2005; Li et al., 2012). As light levels increase, released dopamine acts on D_4_Rs in photoreceptors to decouple them, transitioning retinal signaling from rod to cone dominance (Derouiche and Asan, 1999; Ribelayga et al., 2008; Jin et al., 2015).

Dopamine similarly reduces coupling in the retinal network. Horizontal cell homologous coupling is reduced by dopamine and by light in a variety of species (Dong and McReynolds, 1991; Xin and Bloomfield, 1999; Packer and Dacey, 2005), which reduces the receptive field size of horizontal cells. The functional significance of this well-known fact has not been clearly understood. One plausible example is that horizontal cells contribute to the receptive field surround of a subset of primate ganglion cells, and dopamine modulates the surround inhibition to those ganglion cells (Mcmahon et al., 2004; Zhang et al., 2011) [but see mouse ganglion cells (Dedek et al., 2008)]. The homologous coupling of AII amacrine cells is also regulated by dopamine. AII amacrine cells are a critical component of the rod-signaling pathway, and accordingly, dopamine works as it does in rod photoreceptors. Individual AIIs can pass small signals within a wide network of AIIs through gap junction coupling in low light conditions, whereas this coupling is closed by light. In this way, dopamine in bright ambient conditions contributes to the dominance of cone-mediated signaling (Pourcho, 1982; McMahon and Mattson, 1996; He et al., 2000; Zhang et al., 2011; Hirasawa et al., 2012).

In addition to its effects on gap junctions, dopamine also reduces the response gain across cell populations, preventing saturation as ambient light levels increase. The gain control by retinal dopamine is primarily mediated by modulating voltage-gated channels, similar to its effects seen elsewhere in the CNS (Surmeier et al., 1995; Carr et al., 2003; Rosenkranz and Johnston, 2006). At the level of photoreceptors, dopamine works on D_4_Rs to decrease the responsivity of rod photoreceptors by suppressing an I_h_ current required for rod repolarization (Kawai et al., 2011). Dopamine furthermore modulates horizontal cells and subsets of RGCs *via* D_1_R inhibition of voltage-gated Ca^2+^ or Na^+^ currents to decrease visual signaling (Jensen and Daw, 1986; Vaquero et al., 2001; Hayashida and Ishida, 2004; Hayashida et al., 2009; Blasic et al., 2012; Ogata et al., 2012; Liu et al., 2016b). Lastly, previous studies have shown that light-adaptation or activation of D_1_Rs in bipolar cells leads to increased GABAergic feedback from horizontal cells, increasing the strength of surround inhibition (Cook and McReynolds, 1998; Chaffiol et al., 2017). Taken together, dopamine mediates light adaptation in retinal cell populations through gap junction regulation and by controlling neuronal response gain control.

Finally, dopaminergic amacrine cells exhibit a specific connection to the intrinsically photosensitive retinal ganglion cells (ipRGCs) that are key neurons for the circadian rhythm *via* their entrainment of the suprachiasmatic nucleus (Berson et al., 2002; Qiu et al., 2005). Dopaminergic cells receive excitatory retrograde visual signaling from M1 ipRGCs (Zhang et al., 2012; Prigge et al., 2016). In turn, released dopamine regulates melanopsin mRNA expression in ipRGCs and reduces their light responses (Sakamoto et al., 2005; Van Hook et al., 2012). In this way, dopamine also appears to modulate the maintenance of circadian rhythms.

### A Novel Role for Dopamine in Retinal Signal Processing: Visual Signal Decorrelation

As we described, the olfactory bulb and retina exhibit various similarities. Both systems utilize comparable neural networks to process incoming receptor signals before projecting to the cortices. Moreover, both systems contain neuromodulators that tune signal processing. One such modulator is dopamine, which coordinates D_1_R and D_2_R signaling among neurons residing in each system. However, several dissimilarities are also present. In the olfactory bulb, dopamine plays a role in odor signal decorrelation. In the retina, dopamine plays a role in light adaptation and circadian rhythm; however, visual signal decorrelation has not previously been attributed to dopamine modulation. Our findings in the present study demonstrate a novel role for dopamine in the retina, increasing the peak temporal tuning of some bipolar cell types, and regulating temporal overlap between bipolar cell types.

In the retina, different features of images such as color, motion, and shape are encoded through distinct neural pathways, which are sent out to the brain in parallel (Enroth-Cugell and Robson, 1966; Boycott and Wässle, 1974; Livingstone and Hubel, 1987, 1988; Awatramani and Slaughter, 2000; Wässle, 2004). Parallel processing starts as early as the second-order neurons in the retina, bipolar cells, which extract distinct features of image signaling from photoreceptors and encode them across approximately 15 types of bipolar cells (Wu et al., 2000; Ghosh et al., 2004; Wässle et al., 2009; Helmstaedter et al., 2013; Shekhar et al., 2016). Distinct functions for each type have been gradually understood (Euler et al., 2014). Type 1 through 4 bipolar cells are classified as OFF bipolar cells, encoding light offset, while types 5–9 and rod bipolar cells (ON-bipolar cells) encode the onset of light. Rod and cone signaling is also encoded through distinct types of bipolar cells. Additionally, chromatic information is also uniquely separated, where bipolar cells types 1 and 9 carry mid- and short-wavelength light information, respectively (Breuninger et al., 2011). Furthermore, each bipolar cell type exhibits distinct temporal tuning. Types 2, 3, 5, 7 bipolar cells encode fast-changing light stimuli (e.g., object motion, object edge detection), while the others likely encode more stationary features of light (e.g., color, shape; Ichinose et al., 2014; Ichinose and Hellmer, 2016). Previously, we found that D_1_Rs are expressed by bipolar cells in a type-dependent manner (Farshi et al., 2016). If dopamine modulates visual signal processing only in a subset of bipolar cells, this will demonstrate temporal signal decorrelation, a new role of dopamine in the retina.

We found that a D_1_R agonist, SKF38393, shifted peak frequency responses towards higher frequencies in a subset of bipolar cells (Figures 3, 4). In ON-bipolar cells, D_1_Rs are expressed by type 5-2, XBC, 6, and 7 bipolar cells (Farshi et al., 2016), which exhibit mid to high-frequency tuning (Ichinose et al., 2014). We found that SKF increased the peak frequency responses in type 5, 6, and XBC that were consistent with the D_1_R-expressing bipolar cell types. There are no known markers for subsets of type 5 bipolar cells, and thus, we were not able to confirm that type 5 cells we recorded were D_1_R-expressing types. However, we found that the frequency tuning of SKF-sensitive cells was higher than that of the SKF insensitive cells (Figure 4). Taken together, our data suggest that dopamine enables high-temporal tuning ON-bipolar cells to respond to higher frequencies through D_1_Rs.

For a subset of OFF bipolar cells, dopamine also shifted the peak frequency response to higher frequencies (Figures 3, 4). In the present study, we had only a limited number of OFF cells for each type and could not correlate morphological types and SKF sensitivity. However, only a subset of OFF bipolar cells was SKF-sensitive, suggesting type-specific dopaminergic modulation. We previously showed that D_1_Rs are expressed by type 1, 3b, and 4 OFF bipolar cells which are low-frequency tuning cells (Farshi et al., 2016; Ichinose and Hellmer, 2016). Therefore, this suggests that dopamine may boost the temporal response of previously low-frequency tuning bipolar cells towards higher frequencies, such that all OFF bipolar cells would become sensitive to higher frequencies in contrast to the ON pathway bipolar cell types.

To explore the underlying mechanisms, we examined the dopaminergic effect on voltage-gated Ca^2+^ currents and HCN currents. HCN currents were increased by SKF in a subset of bipolar cells (Figure 5). HCN currents are critical for rhythmic activities in the heart pacemaker cells and neurons in the central nervous system (Baker et al., 1997; Day et al., 2005; Knop et al., 2008). In contrast, in the retina HCN channels have been associated with transient signaling as well as higher-frequency tuning (Cangiano et al., 2007; Della Santina et al., 2012; Puthussery et al., 2013; Bemme et al., 2017). These results suggest that HCN currents shape the high-frequency responses. Voltage-gated Ca^2+^ currents were examined because it may increase the membrane excitability. Contrary to our expectation, LVA currents were reduced by SKF in a subset of bipolar cells (Figure 6). LVA Ca^2+^ currents support the burst spiking activity in neurons (Fan et al., 2000; Pellegrini et al., 2016); however, its effect on graded synaptic responses has not been understood. Future investigation will need to elucidate the mechanism of temporal response modulation in bipolar cells by dopamine.

Alternatively, the observed effects of SKF could come from other D_1_R containing neurons. While we could not directly rule out possible contributions from D_1_Rs in other neurons such as AII amacrine or horizontal cells, we minimized this possibility through our experimental conditions. For example, we adapted the preparations with a rod-saturating light background before recordings; therefore, D_1_R-mediated AII amacrine or horizontal cell uncoupling and amplitude reductions likely were already present before recording. Moreover, the 100 μm diameter spot size of the stimulus would likely be small enough to mitigate horizontal cell feedback that was not already blocked by bicuculline. Interestingly, AII-AII gap junction coupling within the AII network can act as a low-pass filter (Veruki and Hartveit, 2002; Veruki et al., 2008) in a similar frequency range to signals observed in this study. However, it has been shown that primarily sustained currents pass between cone bipolar cells through the AII network (Kuo et al., 2016), which would be unlikely to affect the change in high-frequency tuning that we observed. Furthermore, it does not explain why a subset of ON bipolar cells was SKF-insensitive. Therefore, we concluded that our results of dopaminergic temporal response modulation were attributable to direct bipolar cell activation.

Inspired by a comparison between the olfactory bulb and the retina, we found that a general function of dopamine is to mediate signal decorrelation in both systems, despite using unique mechanisms in each. Ultimately, this study adds to a growing body of evidence that the intrinsic signaling properties regulating bipolar cell output are subject to extrinsic tuning both *via* inhibition as well as neuromodulation (Ayoub and Matthews, 1992; Tooker et al., 2013; Franke et al., 2017; Hall et al., 2019) to shape parallel signal processing in the retina.

## Data Availability Statement

Requests to access the datasets should be directed to tichinos@med.wayne.edu.

## Ethics Statement

The animal study was reviewed and approved by the Institutional Animal Care and Use Committee at Wayne State University (protocol no. A05-03-15).

## Author Contributions

TI designed the study, collected experimental data, and performed statistical analysis. CH, JB, LH, CK, and TI wrote the manuscript. All authors contributed to the article and approved the submitted version.

## Conflict of Interest

The authors declare that the research was conducted in the absence of any commercial or financial relationships that could be construed as a potential conflict of interest.
